# Maternal toxoplasmosis and the risk of childhood autism: serological and molecular small-scale studies

**DOI:** 10.1186/s12887-021-02604-4

**Published:** 2021-03-17

**Authors:** Jamila S. Al Malki, Nahed Ahmed Hussien, Fuad Al Malki

**Affiliations:** 1 Department of biology, College of Science, Taif University, P.O. Box 11099, Taif, 21944 Saudi Arabia; 2 Pediatric Department, College of Medicine, Majmaah University, P.O. Box 66, Almajmaah, 11952 Kingdom of Saudi Arabia

**Keywords:** Toxoplasmosis, Autism, IgG, IgM, Nested PCR, RFLP

## Abstract

**Background:**

Toxoplasmosis resulting from infection with the *Toxoplasma* parasite has become an endemic disease worldwide. Recently, a few studies have reported a high prevalence of Toxoplasmosis infections among Saudi Arabian women. This disease could become life threatening for pregnant women and for immunodeficient people. There is evidence that infections during pregnancy, especially in the early stages, are associated with neurodevelopmental disorders. Autism disorder represents one of the most common neurodevelopmental disorders worldwide; it is associated with delayed language development, weak communication interaction, and repetitive behavior. The relationship between prenatal toxoplasmosis and autism in childhood remains unclear. The present study aims to report a link between maternal toxoplasmosis and autistic offspring among Saudi Arabian women.

**Method:**

Blood samples (36 maternal, 36 from their non-autistic children, and 36 from their autistic children) were collected for serological and molecular evaluation.

**Results:**

A toxoplasmosis infection was reported for 33.34% of participants using an ELISA assay (5.56% IgG+/IgM+, 11.11% IgG−/IgM+, and 16.67% IgG+/IgM-); however, a nested PCR assay targeting *B1* toxoplasmosis specific genes recorded positive tests for 80.56% of the samples. In addition, the present study detected several points of mutation of mtDNA including NADH dehydrogenase (*ND1*, *ND4*) and *Cyt B* genes and the nDNA pyruvate kinase (*PK*) gene for autistic children infected with toxoplasmosis.

**Conclusion:**

Considering previous assumptions, we suggest that a maternal toxoplasmosis infection could have a role in the development of childhood autism linked to mtDNA and nDNA impairment.

## Background

*Toxoplasma gondii* (*T. gondii*) is an infectious protozoan that invades warm-blooded animals including humans and leads to a globally widespread disease known as toxoplasmosis [1]. Toxoplasmosis can be life threatening especially for pregnant women and for immunodeficient individuals (cancer and HIV+ patients and organ transplant recipients) [2]. Among Saudi women of reproductive age, there was an approximately 27.8% *T. gondii* seroprevalence from 2000 to 2017 [3].

The main transmission modes of the parasite are raw or undercooked meat, contaminated water, and cat feces [4, 5]. Although no symptoms may appear in adults or children after direct transmission of the parasitic infection, there are nonetheless dangerous effects from congenital transmission through the placenta of pregnant women to their fetuses that can affect the central nervous and muscular systems [4, 6].

A few studies have reported a relationship between maternal toxoplasmosis serologically and the risk for autistic offspring [7, 8]. Fond et al. [9] suggested that *T. gondii* tachyzoites may invade different types of brain cells in the cerebellum that in turn control signaling pathways and signal transduction mechanisms that are involved in many functions including cell apoptosis, immune cell maturation, and antimicrobial effector functions. Wang et al. [10] linked the parasite with the apoptosis induction of neural stem cells via a stress pathway in the endoplasmic reticulum.

To date, no clear epidemiological link has been established between toxoplasmosis and autistic children except for a few recent reviews suggesting that the biochemical disturbances and brain morphological findings in autism are associated with *T. gondii* infections [11–13].

Autism is defined as a neurodevelopmental disorder that affects the ability of adults and children to communicate, to interact socially, and to respond to specific stimuli in their surroundings. In addition, autism disorder is characterized by delayed language development, repetitive patterns of behavior, and difficulties with social imagination and interaction [14].

The present study aimed to assess maternal infection with toxoplasmosis serologically and then by nested PCR to report infections in their autistic children and to correlate their infections and autism using specific related genes.

## Methods

This prospective study combines qualitative (questionnaire for mothers and their children) and quantitative (serological and molecular) data and investigates whether or not they are consistent.

### Participant selection

We have selected participants from interviews with autistic children’s families; however, the participants did not know whether or not they were infected with toxoplasmosis. It is relatively small sample size study that is limited to participants according to interviews with families. In which 108 blood samples were drawn from maternal (Mo), non-autistic (N) and autistic (A) child participants in 36 families. The sample size was determined according to statistical recommendations and other previous study that had used samples from only 15 autistic patients versus 13 control, also a relatively small sample size, but it represents a limitation to studies involving postmortem brains [15]. The autistic children in the present study were not hospitalized and had previously been diagnosed with autism by specialists in government hospitals in Jeddah, KSA.

### Maternal/autistic child questionnaires

Questionnaire was developed for this study only and was sent to mothers to familiarize themselves with its contents before they decided to enroll in the study. Two questionnaires were answered by mothers: one about themselves and the other about their autistic offspring.

Maternal Questionnaire: The questions elicited basic information including the number of non-autistic and autistic children in the family and their ages and birth orders while others were related to maternal health status including disease history, previous spontaneous miscarriages and illnesses during pregnancy with her autistic offspring. Others related to lifestyle such as having a pet like a cat in the home, dealing with raw/uncooked meat, and frequent travel to remote deserted areas. The questionnaire also asked about any previous diagnosis with toxoplasmosis and the incidence of autism among relatives.

Questionnaire about autistic offspring: Again, basic information was sought in addition to questions on their health status before and after diagnosis with autism and any previous diagnosis with toxoplasmosis.

### Sample collection

Blood samples were drawn by a licensed nurse (License number: 2600046728) from participants in their homes. In all, a 5 ml blood sample was drawn from each participant using one syringe for each one. The sample was then divided into two separate tubes, one without EDTA for a serological assay and the other with EDTA for a molecular assay. The tubes were kept at 4 °C for further evaluation. All medical wastes were discarded as biohazardous waste by the company SEPCO, Jeddah, KSA.

### Serological evaluation

According to the Sunlong Biotech® instruction manual (China), EDTA-free blood samples were centrifuged at 2500 rpm for 20 min then collected sera were used to detect Toxo-IgG and Toxo-IgM using sandwich-ELISA, separately. Briefly, 50 μl of diluted serum in a dilution buffer (1:4) were added to micro-ELISA strip plate wells (separately pre-coated with an antigen specific to Toxo-IgG or Toxo-IgM) that in turn combined with their specific antigen. Wells were gently shaken, incubated at 37 °C for 30 min and then washed with a washing buffer (5 times). The Horseradish Peroxidase (HRP)-conjugate reagent was added to the wells, incubated, and then washed. Finally, chromogen solutions were added for color development and incubated at 37 °C for 15 min. The reaction was terminated by adding 50 μl of stop solution to each well. The plates were read at 450 nm optical density using spectrophotometry. We left an empty well as a blank control and two wells as negative and positive controls supplied with the kit. The presence of Toxo-IgG or Toxo-IgM was determined by separately comparing data with their cut-off values. The critical value (cut-off) was calculated as the average value of negative control + 0.15, i.e., a negative OD value < cut-off while a positive OD value ≥ cut-off.

### Molecular evaluation

#### Nuclear DNA (nDNA) and mitochondrial DNA (mtDNA) extraction

EDTA blood samples (0.5 ml) were mixed with a 0.5 ml of TKM1 buffer (100 mM Tris-HC1, pH 7.4, 250 mM sucrose, 10 mM EDTA), shaken until the blood changed to a bright red color (due to hemolysis), and then centrifuged at 4000 rpm for 10 min at 4 °C to obtain a nuclear pellet. Supernatant contained mitochondria was collected in a fresh microcentrifuge tube, then both the nuclear pellet and mitochondrial supernatant residuals were centrifuged at 10,500 rpm for 10 min at 4 °C. Nuclear and mitochondrial pellets were suspended in 480 μl of a high-salt buffer (Tris HCl 10 mM pH 7.6, 10 mM KCl, 10 mM MgCl_2_, 0.4 M NaCl and 2 mM EDTA), 75 μl of 10% SDS and 1 μl Proteinase k (10 mg/ml) and incubated at 55 °C for 30 min. Protein was removed by salting out, and then nDNA and mtDNA were precipitated separately using cold ethanol alcohol and were then dissolved in sterile distilled water [16].

### Nested PCR

Two different specific primers were used for the first round of PCR (FP1: 5′- GGAACTGCATCCGTTCATGAG-3′; RP1: 5′-TCTTTAAAGCGTTCGTGGTC-3′) and for the second round (FP2: 5′-TGCATAGGTTGCAGTCACTG-3′; RP2: 5′-GGCGACCAATCTGCGAATACACC-3′) to amplify fragments of *B1* genes to detect *T. gondii* [17]. The PCR reaction mixture (20 μl) was 6 μl sterile distilled water, 2 μl (100 ng/1 μl) extracted nDNA/first PCR product (for the first and second rounds, respectively), 1 μl forward primer (20 pmole), 1 μl reverse primer (20 pmole) and then 10 μl 2x master mix (Promega, USA) in 0.2 ml PCR eppendorf. Expected PCR products from the first and second rounds were 198 bp and 97 bp, respectively.

Cycling began in a thermal cycler with an initial denaturation at 94 °C for 5 min followed by 30 cycles of DNA denaturation at 94 °C for 30 s, primer annealing at 50/56 °C (for the first and second rounds, respectively) for 30 s, primer extension at 72 °C for 30 s, and then final extension at 72 °C for 10 min. All PCR products were separated by 2% ethidium bromide stained-agarose gel and visualized under a UV illuminator according to Sambrook et al. [18].

### Restriction fragment length polymorphism (RFLP)

PstI (10 U/μL, Thermo Scientific, UK) restriction enzyme was used to digest extracted mtDNA to detect points of mutation. PstI cut mtDNA at two different sites at 6914 bp and 9024 bp to produce two bands sized 14,458 bp and 2110 bp. According to pamphlet instructions, digestion reaction was setup by using 5 U of enzyme and then fragments of digested mtDNA were separated using a 2% ethidium bromide-stained agarose gel.

### PCR amplification of autism related genes, sequencing, and alignment

Mitochondrial NADH dehydrogenase (*ND1*, *ND4*) and *Cyt B* genes were amplified from mtDNA to give amplicons sized 69 bp, 84 bp, and 89 bp, respectively, while the pyruvate kinase (*PK*) gene was amplified from nDNA to give a PCR product of 84 bp. The primers used are listed in Table 1, and the PCR reaction program was 10 min at 95 °C, followed by 40 cycles of 15 s at 95 °C, 60 s at 60 °C, and 30 s at 72 °C. The final extension was at 72 °C for 10 min as in Gu et al. [19]. The PCR products were separated at 4% in a low-melting agarose gel in a TBE buffer for about 90 min (at − 4 °C and 100v to prevent band degradation).

**Table 1:** Selected used Primers [19]

Primer	Sequence
Forward *PK*	5′-AGCCCAAATGGCCTTGAA-3′
Reverse *PK*	5′-AGAGACAGAATGCCAGTGAGC-3′
Forward *ND1*	5′-CCCTAAAACCCGCCACATCT-3′
Reverse *ND1*	5′-GAGCGATGGTGAGAGCTAAGGT-3′
Forward *ND4*	5′-CCATTCTCCTCCTATCCCTCAAC-3′
Reverse *ND4*	5′-CACAATCTGATGTTTTGGTTAAACTATATTT-3′
Forward *Cyt B*	5′-CACGATTCTTTACCTTTCACTTCATC-3′
Reverse *Cyt B*	5′-TGATCCCGTTTCGTGCAAG-3′

The PCR products were subjected to sequencing using an ABI Prism 3730 Genetic Analyzer automated sequencer to report the site of mutations. Sequenced PCR products for autistic samples were aligned with normal control sequences using online NCBI Nucleotide BLAST alignment analysis to detect points of mutation.

## Results

### Maternal/autistic child questionnaires

Mothers’ current ages ranged from 35 to 49 years; about 25% of mothers had had a spontaneous abortion for one time and 8% for triple times before or after the birth of her autistic children. The age difference among non-autistic and autistic children in the same family ranged from 1 to 17 years. While the birth order of autistic children among their siblings differed from family to family, after puberty the behavior of elder autistic daughters became somewhat normal according to their families’ observations. None of the participants had received a toxoplasmosis diagnosis before; they had no idea whether they were infected or not. Very few frequently traveled to remote deserted areas (only 25% travel non-continuously) or had direct contact with uncooked meat. The common symptoms among autistic children were pronunciation difficulties, hyperactivity, echolalia, attention deficit, and poor visual communication. Other medical conditions included the presence of a fluid sac behind the ear of one autistic child that when surgically removed improved the child’s behavior, congenital heart defects in another. Only two families reported one or more relatives with autism. Only a few of the autistic children had undergone any behavior therapy, others took Omega-3 and Atomoxetine, and a few had both Atomoxetine and behavior therapy. The rest were untreated.

### IgG, IgM and nested PCR diagnosis of toxoplasmosis

**Fig. 1:**
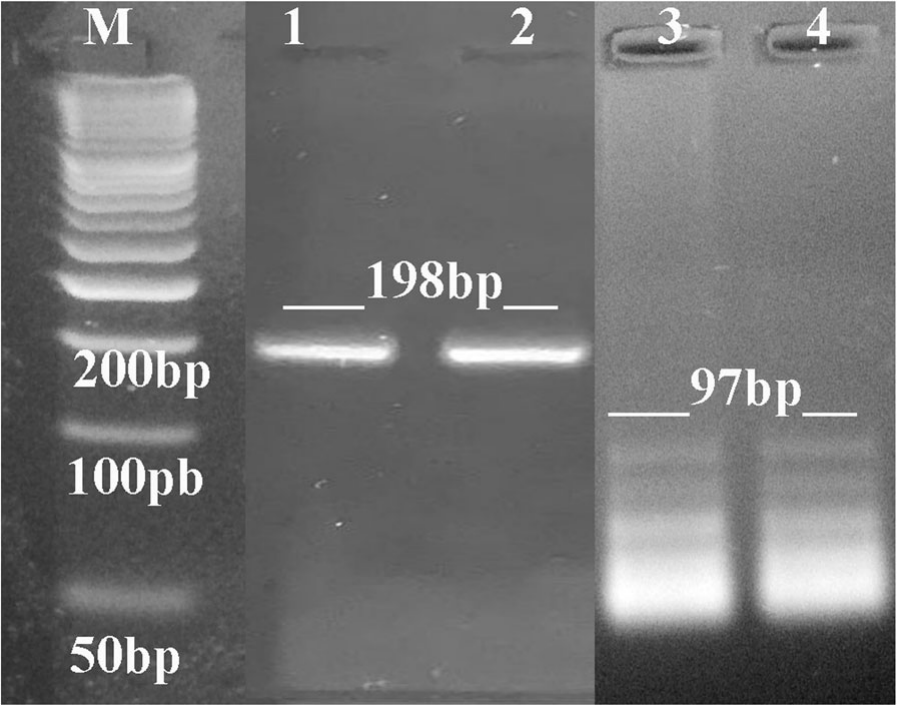
Representative agarose gel (2%) showing PCR product for 1st and 2nd rounds to detect 198 bp and 97 bp of B1 gene specific to Toxoplasmosis, respectively. In which, M represents low molecular weight DNA marker (50-1500 bp), lanes 1 & 2 represent first round and lanes 3 & 4 represent second round of nested PCR, however it shows other lower non-specific bands

Of the 108 sera samples diagnosed serologically for the presence of toxoplasmosis IgG and/or IgM, 6 Mo samples (5.56%) had positive IgG and positive IgM, 12 (11.11%) (3Mo and 9A) had negative IgG and positive IgM, and 18 (16.67%) (6 N and 12A) had positive IgG and negative IgM. A total of 72 samples (66.67%) showed negative IgG and IgM. Therefore, we use nested PCR for the B1 gene that is specific to *Toxoplasma* to ensure the serological evaluation (Table 2). The first round of PCR produced amplicons with a size of 198 bp for all samples of IgG+/IgM+, IgG−/IgM+, IgG+/IgM-, and 51 samples (47.2%) of IgG−/IgM- (24Mo, 18 N and 9A); therefore the PCR detected toxoplasmosis antibodies in a total of 87 (80.56%) of the 108 samples **(**Table 2, Fig. 1**)**, 12 of these samples, represented 100% of female A participants, though 21 (19.4%) samples (3Mo, 18 N and 6A) showed negative PCR products and IgG−/IgM-. Nested PCR (in the second round) confirmed the positive results of the first round by producing PCR products of 97 bp specific to the *B1* gene of *Toxoplasmosis gondii,* as shown in Fig. 1.

**Table 2:** Combined results showing number, age of participants, serological and nested PCR for *Toxoplasmosis gondii* detection for all samples. In which, Mo, mothers; N, non-autistic children and A, autistic children

Groups	Number of Participants	Age range (Years)		Samples with negative results of nested PCR B1 gene (−)	Samples with positive results of nested PCR B1 gene (+)
**Mothers (Mo)**	36	35–49		21 samples (19.4%) (3Mo, 12 N & 6A) All have negative results IgG−/IgM-	- 6 samples (5.56%) (6Mo) with IgG+/IgM+ - 18 samples (16.67%) (6 N & 12A) with IgG+/IgM- - 12 samples (11.11%) (3Mo & 9A) with IgG−/IgM+ - 51 samples (47.2%) (24Mo, 18 N & 9A) with IgG−/IgM-
**Non-autistic children (N)**	36	5–23	Age difference between non-autistic and autistic children in the same family ranges from 1 to 17 years		
**- Gender**	(18 male & 18 female)				
**Autistic children (A)**	36	3–17			
**- Gender**	(20 male & 16 female)				
**Total**	**108**			**21 (19.4%)**	**87 (80.56%)**

### RFLP

**Fig. 2:**
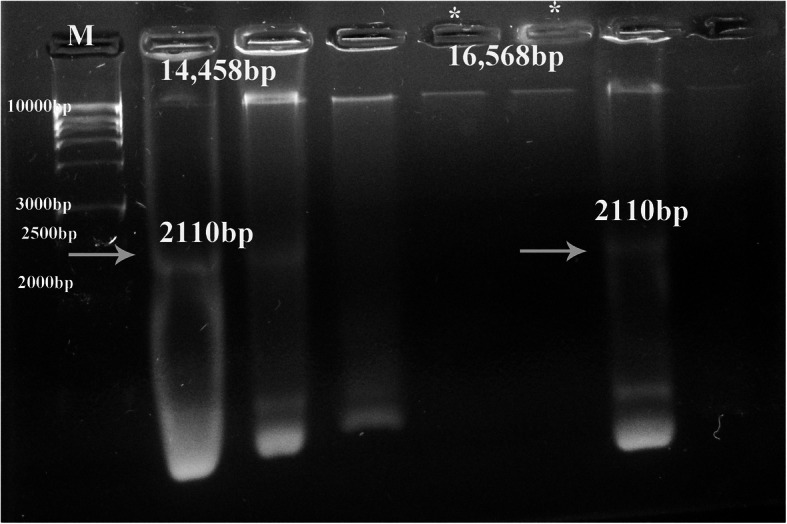
Representative agarose gel photo (2%) showing treatment of mtDNA with restriction enzyme PstI, in which positive cut samples exit 2 bands at 14,458 bp and 2110 bp, while samples cut in one site only (*) gives one band at 16,568 bp only. M refers to high molecular weight marker (250–10,000 bp)

The present study used PstI to detect point of mutation of mtDNA. There are two different sites in mtDNA that are recognized by PstI enzyme, one at 6910-6915 bp (located within ATP6 gene site) and the other site at 9020-9025 bp (located within COX1 gene site). All maternal (Mo), most of the non-autistic (N) and autistic (A) children’s samples were cut by PstI (at both sites) gave two bands at 14,458 bp and 2110 bp. However, 15 samples [12 (A) and 3 (N)] mtDNA gave only one band at 16,568 bp by PstI treatment indicating that those samples were cut in one site (opened linear mtDNA) due to the point of mutation in one of both sites **(**Fig. 2**)**.

### Detection of autism related genes’ mutations

**Fig. 3:**
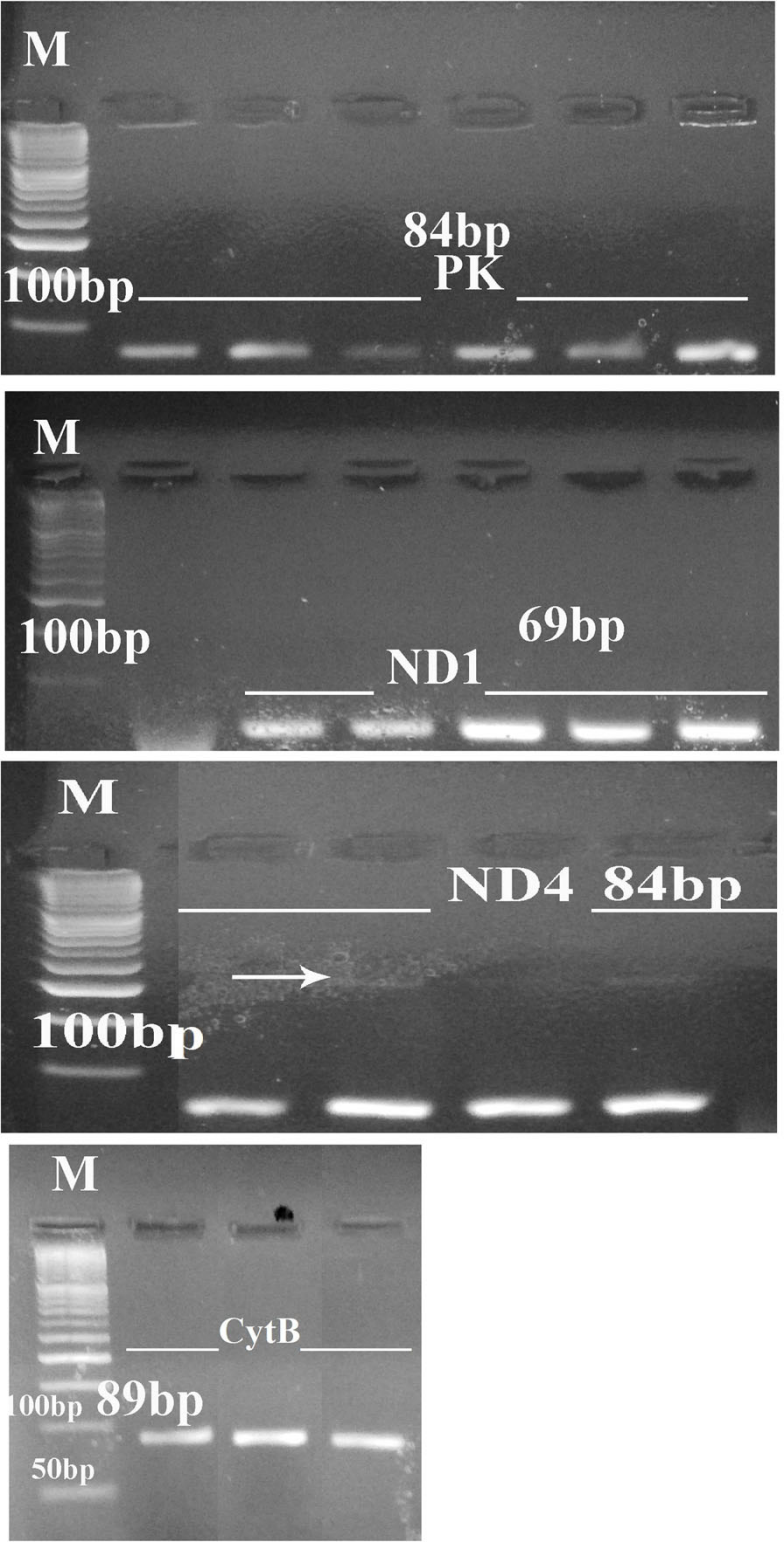
Low melting agarose gel (4%) separates Cyt B (84 bp), ND1 (69 bp), PK (84 bp) and ND4 (84 bp). M refers to low molecular weight DNA marker (50–1500 bp), arrow refers to non-specific additional higher band production

mtDNA was used to amplify three separate genes—*ND1*, *ND4* and *Cyt B*—and nDNA was used to amplify the *PK* gene to yield amplicons of 69 bp, 84 bp, 89 bp, and 84 bp, respectively. Our results report the success of PCR amplification for all selected fragments as shown in Fig. 3.

**Fig. 4:**
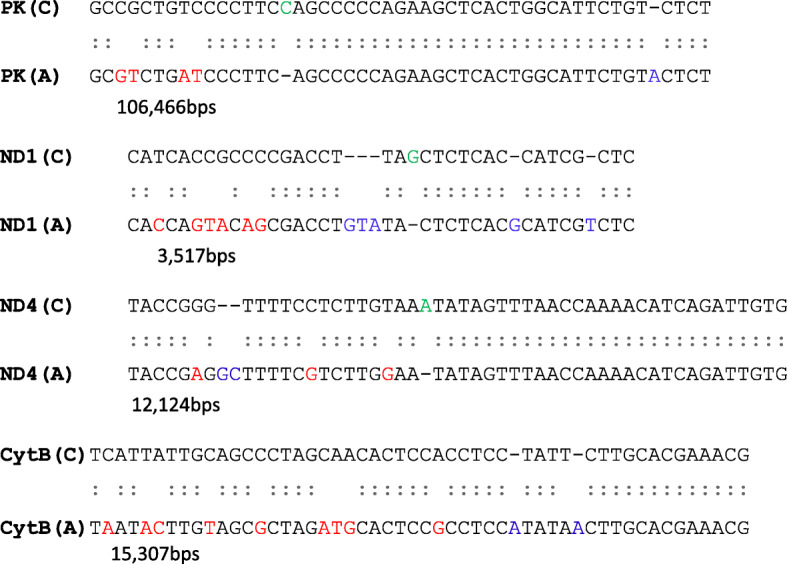
Alignment of DNA sequence of *PK*, *ND1*, *ND4* and *Cyt B* genes’ fragments between control (C) and autistic (A) child using online NCBI Nucleotide BLAST alignment analysis. Colored bps represent different point of mutations including substitution (red bps), insertion (blue bps), and deletion (green bps)

Randomly selected PCR products (autistic samples) from those four genes were sequenced by using their forward/reverse primers and were compared with control sequences to detect the site of mutation. By aligning autistic PCR samples with those of the normal group, we report different points of mutation including deletions, insertions, and base pair substitutions **(**Fig. 4**)**. While mutations differ among the samples except for the nuclear *PK* gene, the sequence shows common mutations in two different autistic samples.

## Discussion

Previous studies have reported the prevalence of fetal congenital diseases, spontaneous abortions, and stillbirths related to *T. gondii* infection during pregnancy [20]. The present results report maternal toxoplasmosis infections and those in their offspring by detecting IgG/IgM antibodies and by using nested PCR to detect the *B1* gene. This is consistent with a previous study that diagnosed infection of pregnant Saudi women from the Aseer region with *Toxoplasma gondii* using immunoglobulin IgG/IgM detection by ELISA followed by nested PCR for the *B1* gene to confirm the ELISA assay and detect other samples that have IgG negative results [17].

The ELISA evaluation detected only 33.34% of the antibodies (5.56% IgG+/IgM+, 11.11% IgG−/IgM+, and 16.67% IgG+/IgM-) while the nested PCR assay detected 80.56% that were specific to the B1 toxoplasmosis gene (Table 2). The first round of the PCR produced amplicons with a size of 198 bp for all samples of IgG+/IgM+, IgG−/IgM+, IgG+/IgM- and for 51 samples (47.2%) of IgG−/IgM- (24Mo, 18 N and 9A) all of which was then confirmed in the second round. Eida et al. [21] concluded that acute infections or low titer antibodies due to recently acquired infections are hard to diagnose by traditional techniques such as detecting anti-*Toxoplasma* IgG or IgM; therefore, using PCR as a direct way to detect the parasite represents a powerful, highly sensitive diagnostic tool that can detect even a single tachyzoite. It has been demonstrated that a PCR-based assay to detect *T gondii* DNA with *B1* repetitive sequences is more sensitive than assays for other targeted genes [22]. In addition, Wastling et al. [23] explained that the high rate of PCR positive results for the *B1* gene could be due to the presence of the parasite’s DNA without viable pathogens because a PCR assay depends on the presence of DNA to give positive results without the need for live parasites.

The two major routes of human Toxoplasmosis transmission are oral and congenital through the maternal placenta to her fetus. Parasitic cysts are commonly present in skeletal muscles, the myocardium, and the brains of human tissue [24]; we therefore reported separately on specific symptoms/diseases found in four autistic children in our sample that may be related to congenital toxoplasmosis infection: congenital heart foramina, head swelling, Krabbe disease and a fluid sac behind the ear.

To our knowledge, few research studies have investigated a serological link between maternal *T. gondii* infection and the risk for autistic offspring. Spann et al. [8] reported a relationship between a high level of maternal *T. gondii* IgM antibodies and a decrease in autistic child probability; however, a lower maternal *T. gondii* IgG antibody level had the reverse effect of increasing the probability of autistic offspring. They concluded that there is a relationship between maternal *T. gondii* IgG/IgM antibodies and the probability of having an autistic child that might be related to the immune response to the parasite or to the whole immune system activation of the host. Another prior study reported that newborns derived their *T. gondii* IgG from their mothers and also suggested that higher levels of IgG in the first quartile of pregnancy increased the probability of autism significantly more than in the second and fourth quartiles [7]. This might explain our results about infected *T. gondii* mothers having both autistic and non-autistic offspring with positive IgG and/or IgM results.

Previously, Thong [25] thought that damage to the neocortex, limbic cortex, and primitive striatal complex in the brain of an autistic child could be due to toxoplasmosis infection. Later, it was determined that *toxoplasma* increased dopamine production that in turn might affect human behavior [26] as reported in the autism spectrum disorder [27, 28]. There are similarities in neuropathological changes and clinical appearances between autism and congenital/chronic latent toxoplasmosis that led to the idea that *T. gondii* infections could cause autism development and attention deficit-hyperactivity disorders [12]. In addition, Flegr [29] postulated that *T. gondii* could affect human behavior via several possible mechanisms including increased levels of dopamine and testosterone that in turn could lead to the cellular immunity impairment of the host thus increasing the chances of their survival inside the host organism.

The present study detects points of mutation of toxo-infected autistic mtDNA samples using PstI enzyme at one of both sites that located within ATP6 and COX1 gene sites. In previous study, it was reported that most of mtDNA mutations (about 56%) were related to ATP6 gene, that in turn altering few conserved amino acids to others that could potentially affect ATPase 6 function that is accompanied with autism pathogenesis [30]. Other study detects that amyloidosis in the autism is related to the most frequent variation (about 31%) of COX1 gene [31]. It was reported that brain parasitism with *T. gondii* tachyzoites found in different diseases, included neurodegeneration diseases, changed the stability and degradation of host cell protein, including modulation of brain ATP production by mitochondrial oxidative phosphorylation [32].

Moreover, mtDNA mutation was reported in other different sites by sequencing the NADH dehydrogenase (*ND1*, *ND4*) and *Cyt B* genes for autistic children infected with *T. gondii*. The present results are in consistent with a previous study that reported the *ND1* gene mutation of mtDNA by sequencing sperm cells of men infected with Toxoplasmosis [33]. It was determined that *T. gondii* growth stages used carbon sources (glucose and glutamine) for energy metabolism that were mostly dependent on mitochondrial metabolism and oxidative phosphorylation that was clearly demonstrated in the extraordinary capacity of the parasite to rapidly multiply within a wide range of host cells [34].

Today it is clear that autism is associated with mitochondrial dysfunction that could be due to a primary or secondary mitochondrial disease. Primary mitochondrial disease presents due to defects in mtDNA and/or nuclear genes needed for mitochondrial function that in turn affect proteins participating in oxidative phosphorylation. However, it is not clear whether mitochondrial dysfunction is a cause for autism or an effect of it [35–38].

mtDNA consists of about 37 genes that are needed to code electron transport chain complexes subunits I, III, IV and V [37]. The *ND1*, *ND2*, *ND3*, *ND4*, *ND4L* and *ND6* genes (mitochondrial NADH dehydrogenase genes) encode complex I subunits while the mitochondrial *Cyt B* gene encodes the complex III subunit; however, complex II is coded only by the nuclear genome such as the pyruvate kinase (*PK*) gene. Our results record several points of mutation (substitutions, insertions and deletions) in electron transport chain genes (*ND1*, ND4, *Cyt B* and *PK*) that in turn could be translated into an altered protein or earlier stoppage of protein translation due to a frame shift that could then lead to increasing gene copy numbers. The present results are in agreement with previous studies that record increases in *ND1*, *ND4* and *Cyt B* copy numbers, mtDNA deletions of *ND4* (44%) and *Cyt B* (33%) in the brain’s frontal cortex, and mtDNA mutations or deletions in the muscle tissue of autistic patients [19, 39].

**Fig. 5:**
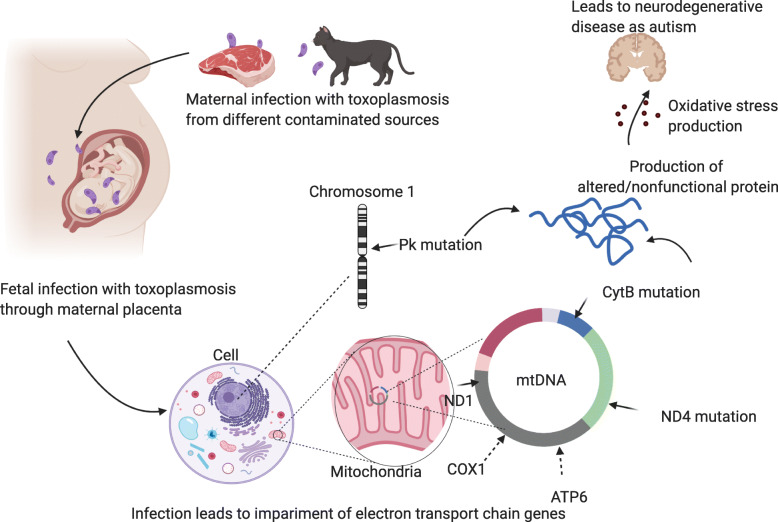
Maternal toxoplasmosis infection leads to impairment of electron transport chain genes of fetus that increases oxidative stress production and in turn leads to autism. Created in Biorender.com

Our assumption is that maternal toxoplasmosis infection from different contaminated sources leads to fetal infection through placenta that depends on infection load and quartile of pregnancy during infection. Accordingly, it is considered that fetal toxoplasmosis infection leads to impairment of electron transport chain genes (complexes I and III) that has an important role in free radical generation and oxidative stress production that in turn leads to the pathophysiology of neurodegenerative and neurodevelopmental disorders such as autism (Fig. 5).

### Limitations

The sample size may have been relatively small, in which all the participants were selected from interviews with families and further larger studies are required to confirm these results. *Inclusion criteria*: Women and their children (autistic and non-autistic) with toxoplasmosis had to be willing and able to provide written, informed consent. *Exclusion criteria*: pregnancy, evidence of a significant, active hematological disease and/or cumulative blood donations during clinical trials in the last three months.

## Conclusions

In conclusion, according to the results of this study, toxoplasmosis could have a role in the development of autism that is linked to mtDNA and nDNA mutations. Therefore, early treatment for maternal *T. gondii* infections could decrease the risk of autism development in their offspring though more samples and different techniques should be used to confirm this assumption. In addition, more research is needed to explain the mechanism of autism development due to toxoplasmosis infection.

## Data Availability

The datasets used and/or analysed during the current study are available from the corresponding author on reasonable request.

## Abbreviations

A: autistic child
HRP: Horseradish Peroxidase
Mo: maternal
mtDNA: mitochondrial DNA
nDNA: Nuclear DNA
N: non-autistic child
*PK*: pyruvate kinase
RFLP: Restriction fragment length polymorphism
*T. gondii*: *Toxoplasma gondii*
